# Exposure to cement industry emissions and respiratory health outcomes: a global systematic review and meta-analysis from 2000 to 2025

**DOI:** 10.3389/fpubh.2026.1887654

**Published:** 2026-09-03

**Authors:** Zahid Ali Khan, AliSalman Sheikh, Łukasz Afeltowicz, Jacek Gądecki

**Affiliations:** 1 Department of Society and Technology Studies, AGH University of Krakow, Kraków, Poland; 2 Medical College, Aga Khan University, Karachi, Pakistan

**Keywords:** cement factory emissions, low and middle income countries, occupational health, respiratory function, respiratory tract diseases

## Abstract

**Introduction:**

Cement manufacturing is a major contributor to industrial air pollution and releases particulate matter and toxic emissions, adversely affecting the respiratory health. Previous studies have reported respiratory impairment and its associated symptoms among exposed populations, yet the evidence remains inconsistent across occupational and residential settings. This systematic review and meta-analysis is aimed to quantify the association between exposure to cement factory emissions and respiratory health outcomes, including lung function impairment and respiratory symptoms, among occupationally and environmentally exposed populations.

**Methods:**

A systematic search was conducted in PubMed, Scopus, Web of Science and Google scholar was conducted for studies published between January 2000 and December 2025. Observational studies assessing exposure to cement dust and respiratory outcomes among workers or nearby residents were included. Primary outcomes included Forced Expiratory Volume in one second (FEV_1_), Forced Vital Capacity (FVC), FEV_1_/FVC ratio, and respiratory symptoms. Random effects model was used to perform meta-analysis using standardized mean differences (SMDs) and risk differences (RDs) with 95% confidence intervals (CIs).

**Results:**

A total of 18 studies were included. Exposure to cement factory dust was associated with significant reductions in FEV_1_ (SMD = −0.71), FVC (SMD = −0.56), and FEV_1_/FVC ratio (SMD = −0.57). Chronic cough was significantly more common among exposed individuals, while other respiratory symptoms showed elevated but non-significant risks. Occupational exposure to cement dust exposure demonstrated greater FEV_1_ impairment than residential exposure.

**Conclusion:**

Cement factory emissions are significantly associated with impaired pulmonary function and increased respiratory morbidity, highlighting the need for improved occupational safety measures and environmental emission controls.

**Systematic review registration:**

https://www.crd.york.ac.uk/PROSPERO/view/CRD420251268913, CRD420251268913.

## 1 Introduction

Over recent years global cement production has increased exponentially, reflecting the expansion of modern infrastructure. In 2024, approximately 4.0 billion metric tons of cement were produced worldwide, with China alone accounting for nearly half of total output and India ranking second among producers (1–3). Besides serving as a cornerstone to the global infrastructure, cement manufacture also stands as one of the most environmentally burdensome industrial sectors: cement manufacturing is responsible for roughly 6–8% of total global greenhouse gas emissions (4). These emissions are largely attributed to energy – intensive kiln processes beginning with the calcination of limestone, where calcium carbonate (CaCO₃) is thermally decomposed into calcium oxide (CaO) and carbon dioxide (CO₂), releasing substantial amounts of CO₂ (5). In addition to greenhouse gases, cement production also emits fine Particulate Matter (PM_2.5_ and PM_10_), nitrogen oxides (NOₓ), sulfur dioxide (SO₂), and respirable crystalline silica, all of which adversely affect air quality and human health (6, 7).

Occupational and environmental inhalation exposures to cement factory emissions pose significant respiratory risks and adverse health outcomes (8). A possible mechanism of particulate mediated respiratory obstruction begins with the deposition of particulates in the lower respiratory tract resulting from immune evasion of the upper respiratory tract. This deposition of particulates results in the activation of alveolar macrophages and oxidative stress pathways to release pro-inflammatory cytokines namely, Interleukin-1β (IL-1 β), Tumor Necrosis Factor-Alpha (TNF-α) (9, 10). Recurrent inflammatory response impairs mucociliary clearance, promoting bronchial epithelial injury, and subsequent airway remodeling. This remodeling of airways increases susceptibility to chronic bronchitis, asthma exacerbations, airflow limitation [decline of Forced Expiratory Volume in one second (FEV_1_) and Forced Vital Capacity (FVC)] and interstitial fibrosis (11–13).

Communities residing near cement factories frequently experience elevated pollution gradients (14, 15). Previous atmospheric dispersion studies have demonstrated high pollutant levels within two kilometers (Km) of emission sources depending on local meteorology and topography (16–18). While there are no universally accepted “safe distance” thresholds, epidemiological studies suggest buffer zones of 3–5 kilometers (Km) from heavy industrial facilities to mitigate chronic exposure risks (19). Low-and middle-income countries (LMICs), often experience exacerbated risks due to rapid, unplanned urbanization and informal residential encroachment on industrial peripheries (20, 21). Furthermore, the use of older kiln technologies, lower-grade fuels, and inconsistent enforcement of emission standards, with higher population densities, significantly increases the public health vulnerability of population living in near cement factories within LMICs (22).

As of today, respiratory diseases remain a leading cause of the global disease burden resulting in high morbidity and mortality worldwide (23). Rigorous and quantitative synthesis of the available evidence is essential to clarify the magnitude of respiratory risks associated with cement factory emissions across a variety of geographic and regulatory contexts. Previous literature has largely been narrative or limited to specific cohorts, preventing robust conclusions on risk patterns at a global level (24, 25). This study therefore aims to systematically quantify the association between cement factory emissions and respiratory health outcomes in people living or working in cement factories, explore exposure-symptom relationships in studies stratified based on their socio-economic status and type of exposure, to assess the methodological quality and heterogeneity of existing studies to inform policy on industrial zoning, and international health guidelines.

## 2 Materials and methods

## 2.1 Objectives

This systematic review and meta-analysis aim to quantify the association between exposure to cement factory emissions and respiratory health outcomes in occupational and residential populations. Specifically, to explore exposure–response relationships, their effect modifiers and assess the methodological quality and heterogeneity of existing studies. The systematic review was conducted according to the Preferred Reporting Items for Systematic reviews and Meta-Analyses (PRISMA)-statement (26) and is registered in PROSPERO (CRD420251268913).

## 2.2 Eligibility criteria

Studies were considered eligible if they assessed exposure to cement dust in relation to lung function parameters (e.g., Forced Expiratory Volume in 1 sec (FEV_1_), Forced Vital Capacity (FVC) and the ratio of FEV_1_ to FVC (FEV_1_/FVC ratio) and/or chronic respiratory symptoms among cement plant workers or individuals living near cement facilities. Both occupational and environmental (residential) exposures were included. A range of study designs was considered, including randomized, non-randomized, and observational studies; however, all included studies were observational (cross-sectional, case–control, or cohort). Eligible studies compared exposed populations with appropriate unexposed control groups. Studies were excluded if they were reviews, case reports, conference abstracts without full text or published in language other than English. Additionally, studies lacking clear exposure assessment or relevant outcomes were also excluded. The inclusion period was restricted to studies published between January 2000 and December 2025.

## 2.3 Search strategy and screening

A comprehensive search for literature till December 2025 was conducted in four bibliographic databases, (PubMed, Web of Science, Scopus and Google Scholar) to identify all the relevant studies. The search strategy included Medical Subject Headings (MeSH) along with the keywords, synonyms and related terms, applied as both index terms and free text words. Search strings for each of the four databases are provided as Supplementary Table 1. Duplicate records were excluded using EndNote (X20.5.1) employing the Amsterdam Efficient Duplication-method and Barmer-method (27) and exported to Rayyan AI (28). The reference list of included studies and related systematic reviews were screened to identify any additional records that have been missed in the initial search. Two reviewers (ZAK and AS) independently screened all potentially relevant titles and abstracts for eligibility and then full articles were screened in duplicates. Conflicts were resolved through discussion between reviewers (ZAK and AS) and through arbitration of a third reviewer (ŁA).

## 2.4 Data extraction and quality assessment

An Excel sheet was prepared to extract key details from all eligible studies. Data collected from each publication included study design setting, inclusion and exclusion criteria, age and sex of participants, number of participants in each group (exposed and unexposed), method of assessment for lung function, distance from the cement factory, reported symptoms, mean with standard deviation (SD), and *p*-values for changes in lung function parameters between different groups. Two reviewers (ZAK and AS) independently performed the methodological assessment of included studies using Joanna Briggs Institute (JBI) Appraisal Checklist (29). Studies were evaluated for eight dimensions, such as the eligibility criteria, exposure measurement, outcome measurement, the identification and control of confounders and statistical analysis. Studies were classified as low risk of bias, if there were minimal concerns regarding validity of results. Moderate risk indicated some concerns, but these were not likely to significantly affect the overall findings. Serious/high risk reflected a substantial concern that could influence the study’s outcomes. In case of insufficient information to assess the risk, domains were classified as no information.

## 2.5 Statistical analysis

Primary outcomes were lung function parameters (FEV_1_, FVC and FEV_1_/FVC ratios) measured through spirometry and chronic respiratory symptoms reported through questionnaire. For each of the given lung function parameters and respiratory symptoms, comparisons were made between the exposed and unexposed groups; where individuals exposed to dust from cement factories irrespective of the mode of exposure (residential or occupational) were considered as exposed and those without the exposure were considered as unexposed. Meta-analysis was performed if original data (expressed in mean ±SD) for each lung function parameter and respiratory symptoms (were available) using Metanalysis Package for R (Metafor) (v4.5.2; R Core Team, 2026) (30). A randomized model for continuous data was adopted, due to possible heterogeneity. The standard mean differences (SMD) with 95% confidence intervals (CIs) for continuous data and Risk Differences with 95% CI for dichotomous data were calculated for each lung function parameter and reported chronic respiratory symptoms where applicable. Standard errors (SE) were converted to standard deviations (SD) to maintain consistency in data presentation. *I*^2^ and Tau^2^ (*τ*^2^) statistics were used to calculate the heterogeneity among studies. Hartung–Knapp (HKSJ) adjustment was applied to continuous data to achieve conservative confidence intervals, mitigating substantial heterogeneity. Subgroup analyses were performed based on World Bank income classification with differences assessed using the Q-test for moderators. Random effect meta-regression analyses were further performed to assess whether income classification justified among-study variability in effect sizes. For all analysis a *p*-value <0.05 was considered statistically significant.

## 3 Results

**Figure 1:**
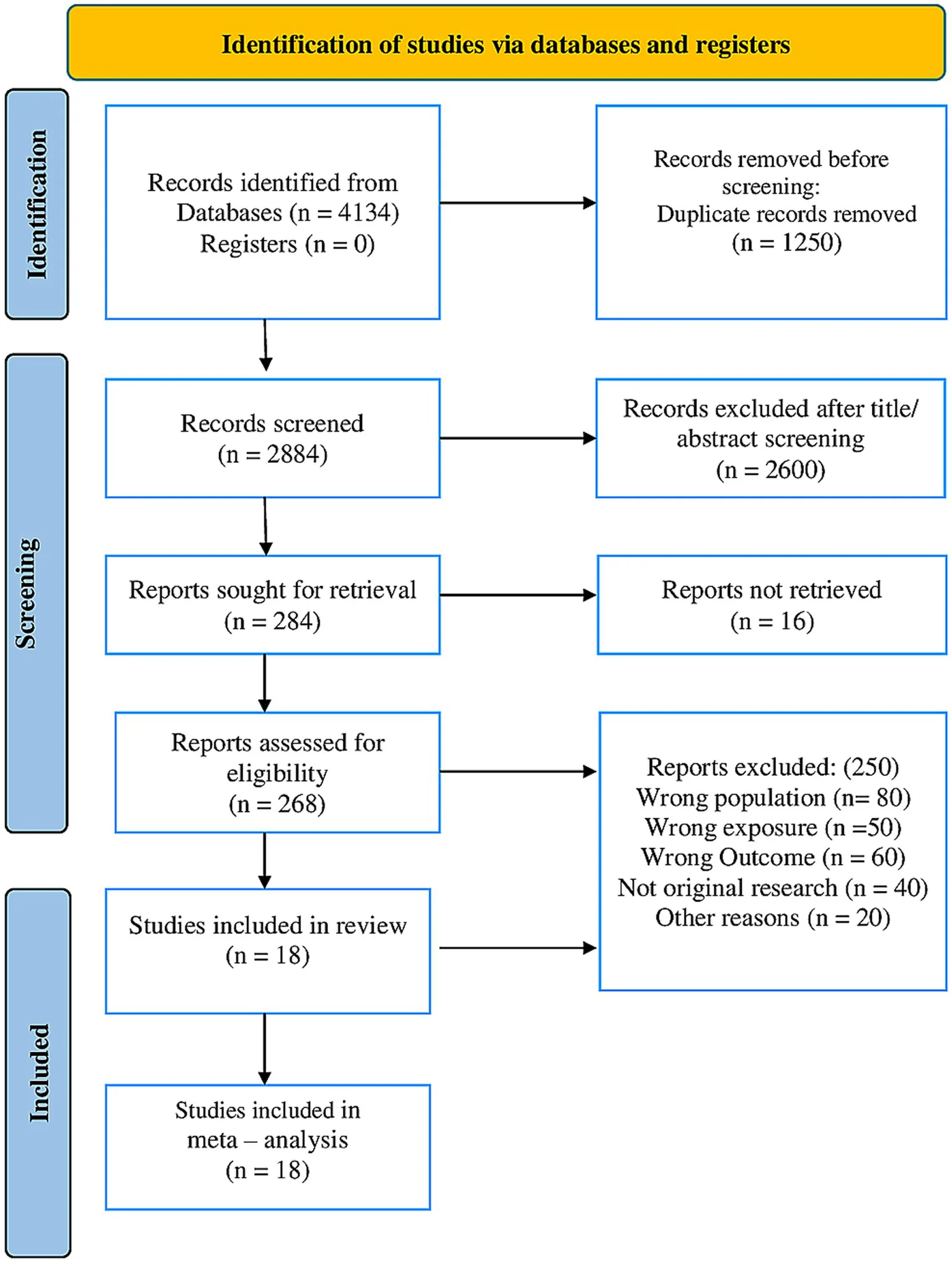
PRISMA flowchart illustrating the study selection process for the systematic review and meta-analysis of the association between cement dust exposure and lung function.

The literature search resulted in a total of 4,134 articles, following removal of 1,250 duplicates, 2,884 titles and abstracts were screened. After screening titles and abstracts, 2,600 studies were excluded, leaving 284 studies for full-text assessment. Of these, 266 studies were excluded due to study design, patient population, outcomes, or language and 18 studies were included in the review (Figure 1).

### 3.1 Study characteristics

This systematic review includes 18 studies published between 2000 and 2025 that were stratified by the type of exposure to dust from cement factories (Table 1). Fifteen studies included occupational type of exposure with participants working in the cement factories. The remaining three studies included participants residing less than 1 kilometer away from cement factories. Twelve studies included male participants only, while two studies (31, 32) did not report sex distribution among its participants, four studies (33–36) included both male and female participants. Studies were also classified based on World Bank Income classification (37), 11 studies were conducted in lower-middle-income countries (LMICs), five studies from upper middle-income countries and two studies from in low-income or less industrialized environments (LIEs). All the studies assessed lung function using spirometry, reporting adherence to standardized protocols of American Thoracic Society (ATS) guidelines, although the manufacturer and model of spirometer and measurement protocols varied across studies. Chronic respiratory symptoms were assessed in 11 studies using structured questionaries from British Medical Research Council (MRC) and ATS. Seven studies reported adjustment for confounders, where all these studies reported adjustment for factors including age, smoking status, body mass index (BMI), socioeconomic status (SES), occupational exposure duration, and work experience. Other less commonly adjusted factors included environmental exposure metrics (e.g., weekly dust hours), biological markers (e.g., blood lead and aluminum levels), and seasonal variation.

**Table 1:** Summary of the characteristics of the 18 studies included in the systematic review, categorized by mode of cement dust exposure (occupational or residential), participant characteristics, World Bank income classification, lung function and respiratory symptom assessment methods, and adjustment for confounding variables.

S/N	Author Year and Country	Sample size (Total)	Type of exposure	Exposure criteria	Exposed group	Unexposed group	Sex (%)	Lung function assessment method	Method for reporting symptoms	Confounders adjusted (Yes /No)	If listed any
Lower Income Countries (LICs)											
1	Zeleke et al. (31) (Ethiopia)	127	Occupational	7.5 h/ day	100	27	NA	SPIRARE spirometer (Cat # SPS310)	Questionnaire	Yes	Age, Height, Smoking status
2	Ali (33) (Sudan)	268	Residential	Exposed = 1.05–1.38 km Unexposed = 4.75–8.02 km	134	134	Male = 44.50 Female = 55.50	Spirometer	Questionnaire (British Medical Research Council)	None	NA
Lower-Middle Income Countries (LMICs)											
3	Dewangan et al. (34) (India)	360	Occupational	Direct dust exposure during 9-h shift	140	220	Male = 96 Female = 4	RMS Helios 702 spirometer	NA	Yes	Age, Smoking status, Duration of service
4	Dushyant et al. (46) (India)	200	Occupational	4–16 h/ day shift for loading and unloading cement bags.	100	100	Male = 100	Easy one Portable spirometer	Questionnaire (British Medical Research Council)	Yes	Age, Smoking, Body Mass Index (BMI) and Socio-economic status
5	Nkhama et al. (35) (Zambia)	118	Residential	Exposed (Freedom) = < 1 km Unexposed (Bauleni) = 18 km	63	55	Male = 52.40 Female = 47.60	Spirometer (NDD Medical Technologies)	Questionnaire	Yes	Sex, Age, Height, Weight, Smoking history and Socio-economic status
6	Aljeesh et al. (32) (Palestine)	100	Occupational	No distance specific given	50	50	NA	Spirometer (Model not specified)	Questionnaire	No	NA
7	Mbelambela et al. (41) (Congo)	379	Occupational	Full shift worker at cement factories for at least five years before study.	223	156	Male = 100	AUTOSPIRO Minato AS-470 spirometer	Questionnaire	NA	Age, BMI, Employment duration, Education, Medical history, Smoking status
8	Merenu et al. (36) (Nigeria)	514	Residential	Two towns (Kalam Baina and Gidan Madi) of Sokoto state in Nigeria as exposed and unexposed groups	244	270	Male = 94.90 Female = 5.10	The Vitalograph Compact (Model 6,600) Spirometer	Questionnaire (American Thoracic Society (ATS))	No	NA
9	Akhter et al. (39) (Bangladesh)	88	Occupational	8–10 h/ day for 6 days/ week for at least 2 years	44	44	Male = 100	CHESTGRAPH (HI-101) Spirometer	NA	No	NA
10	Meo et al. (38) (Pakistan)	100	Occupational	8–10 h/ day 6 days/ week	50	50	Male = 100	The Vitalograph Compact (Model 6,600) Spirometer	NA	No	Age, Height, Weight and Socio-economic status
11	Mwaiselage et al. (67) (Tanzania)	227	Occupational	Personal full-shift (8-h) dust sampling; cumulative exposure in mg/m^3^-years	120	107	Male = 227	Spirometry (Vitalograph spirometer) ATS calibration/BTPS	NA	Yes	Age, pack-years, duration of employment, education
12	Merenu et al. (68) (Nigeria)	152	Occupational	Exposed group included individuals working in cement factory for 6 years	56	96	Male = 152	Vitalograph spirometer Wright peak flow meter (PEFR)	NA	No	Age, sex, height, weight, ethnicity
13	Meo et al. (69) (Pakistan)	100	Occupational	Exposed group included individuals working in cement factories (random selection) and unexposed group were matched local individuals	50	50	Male = 100	Electronic spirometer (Compact Vitalograph UK)	NA	NA	Age, height, weight, socioeconomic status (matched); smoking, comorbidities (excluded)
Upper Middle-Income Countries (UMICs)											
14	Rafeemanesh et al. (45) (Iran)	220	Occupational	Workers from milling and packaging lines with at least 2 years of exposure	100	120	Male = 100	The MIR SPIROLAB III Spirometer	Questionnaire (British Medical Research Council)	No	Age, Smoking status, Seniority
15	Cetintepe et al. (44) (Türkiye)	461	Occupational	Workers from milling and packaging part of production line	263	198	Male = 100	Zan 100 Dry-seal spirometer	NA	Yes	Age, BMI, Experience, Blood aluminum and lead levels
16	Shanshal and Al-Qazaz et al. (43) (Iraq)	194	Occupational	Non-smoking workers from milling and packaging part of production line.	97	97	Male = 100	The MIR SPIROLAB III Spirometer	Questionnaire (British Medical Research Council)	No	NA
17	Thepaksorn et al. (42) (Thailand)	249	Occupational	Exposed group included individuals from production line while unexposed group had office workers	115	134	Male = 100	COSMED SRL portable Spirometer	Questionnaire (ATS) Modified version	Yes	Age, Smoking status, Socio-economic status, Experience, Education level.
18	Hashemi et al. (40) (Iran)	350	Occupational	Exposed group included individuals from production line while unexposed group had office workers	180	170	Male = 100	Spirometer (Model not specified)	Questionnaire	No	Age, Height, Smoking status, Duration of addiction, Work experience

### 3.2 Quality assessment

Across 18 included studies, risk of bias was variable. Fifteen studies assessed using a predefined JBI checklist showed low to moderate overall risk of bias, with three studies showing high risk of bias due to concerns related to confounding, missing data, and outcome measurement. Six studies were rated as having low risk of bias, nine studies were classified as moderate risk, and three studies were judged to have high risk of bias. Overall, a number of studies were methodologically robust, the presence of moderate to high risk of bias in most studies should be considered when interpreting the findings of this review. A detailed domain-level assessment for each study is presented in Supplementary Table 2.

### 3.3 Effect of exposure to dust from cement factories on lung function parameters

**Figure 2:**
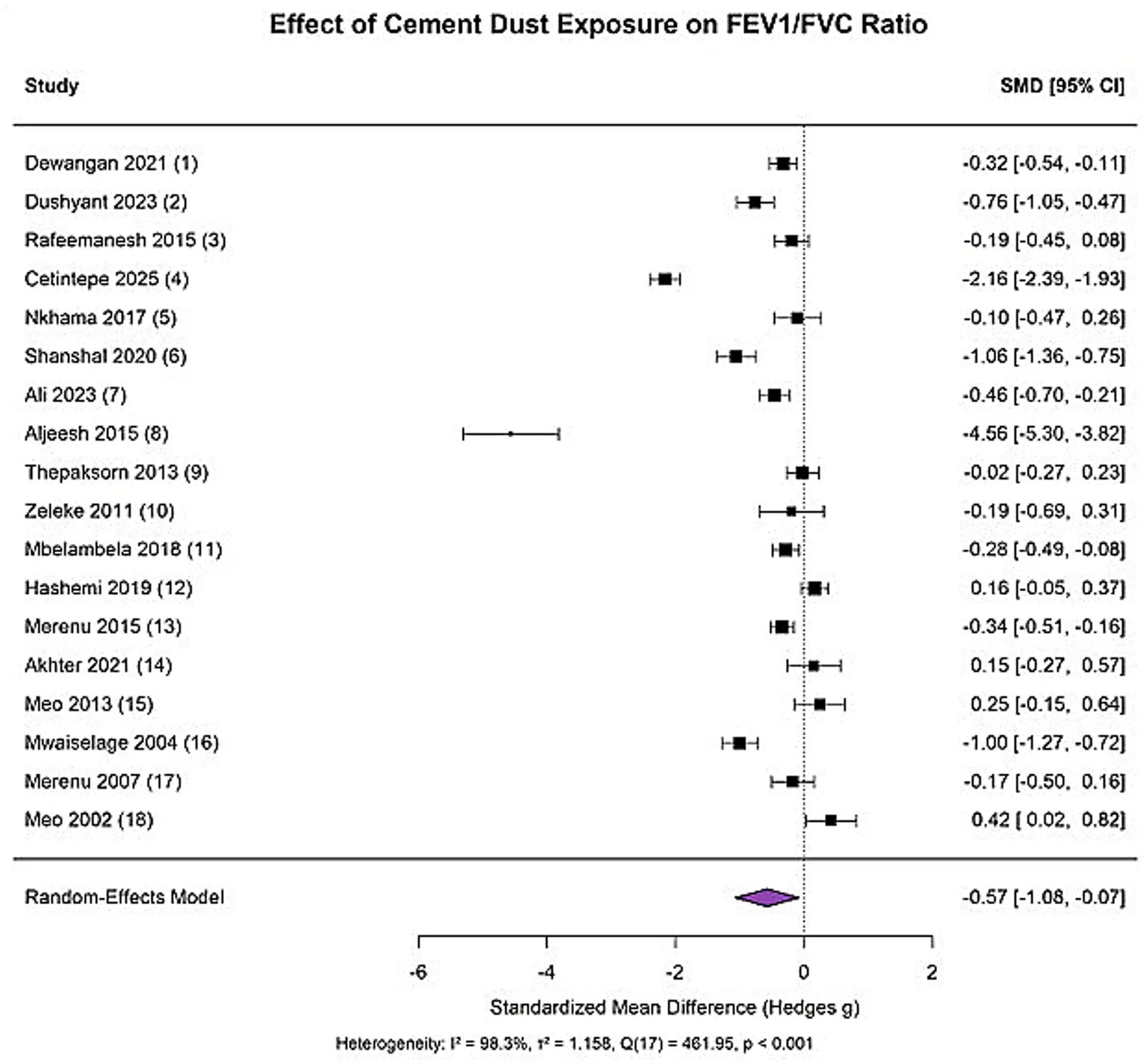
Forest plot of pooled standard mean differences (SMD) for FEV_1_/FVC ratio within groups exposed and unexposed to cement dust from factories across 18 eligible studies.

The metanalysis for effect of exposure of dust from cement factories on lung function parameters (FEV_1_, FVC and FEV_1_/FVC ratio) included 17 studies for FEV_1_ and 18 studies for FVC and FEV_1_/FVC ratio (31–36, 38–47). Dust exposure from cement factories resulted in a statistically significant impact of −0.71 standard deviations (95% CI: −1.05 to −0.37) on FEV_1_ levels among the exposed and unexposed groups (Supplementary Figure 1) and a statistically significant impact of −0.56 standard deviations (95% CI: −0.91 to −0.21) on FVC levels among the exposed and unexposed groups (Supplementary Figure 2). In addition to this, dust exposure from cement factories also resulted in a statistically significant impact of −0.57 standard deviations (95% CI: −1.07 to −0.06) on FEV_1_/FVC ratios (Figure 2 and Table 2).

**Table 2:** Pooled standardized mean differences (SMD) of lung function parameters with corresponding heterogeneity indices and Hartung–Knapp’s (HK) adjusted estimates.

Lung function parameters	Studies (n)	SMD	95% CI	*p*-value	*I*^2^ (%)	*τ* ^2^	SMD (HK)	95% CI (HK)	*p*-value (HK)
FEV_1_	17	−0.713	(−1.052, −0.375)	<0.001	96.23	0.478	−0.713	(−1.091, −0.335)	0.001
FVC	18	−0.564	(−0.917, −0.211)	0.002	96.63	0.557	−0.564	(−0.951, −0.177)	0.007
FEV_1_/FVC Ratio	18	−0.573	(−1.076, −0.069)	0.026	98.34	1.158	−0.573	(−1.131, −0.014)	0.045

### 3.4 Effect of exposure to dust from cement factories on chronic respiratory symptoms

The metanalysis for effect of exposure from dust from cement factories on chronic respiratory symptoms included 9 studies reporting chronic cough (32, 33, 35, 40–43, 45, 46), followed by 7 studies reporting chronic wheeze (33, 35, 41–43, 45, 46) and chronic chest pain (32, 33, 40–42, 45, 46) and 6 studies reporting chronic phlegm (35, 40, 41, 43, 45, 46) (Table 3).

**Table 3:** Pooled risk differences (RD) for chronic respiratory symptoms among individuals exposed to cement dust compared to unexposed controls, based on common effect and random effects (Hartung-Knapp) models.

Chronic respiratory symptoms	Studies (n)	Common effect RD [95% CI]	Random effects (HK) RD [95% CI]	*I*^2^ (%)	*τ* ^2^	*p*-value (HK)
Chronic cough	9	0.17 [0.14, 0.20]	0.28 [0.08, 0.48]	96.7	0.065	<0.001
Chronic phlegm	6	0.06 [0.04, 0.09]	0.15 [−0.01, 0.32]	90.9	0.022	<0.001
Chronic wheeze	7	0.03 [0.01, 0.04]	0.10 [−0.01, 0.20]	85.7	0.009	<0.001
Chronic chest pain	7	0.05 [0.03, 0.07]	0.18 [−0.10, 0.46]	97.5	0.089	<0.001

**Figure 3:**
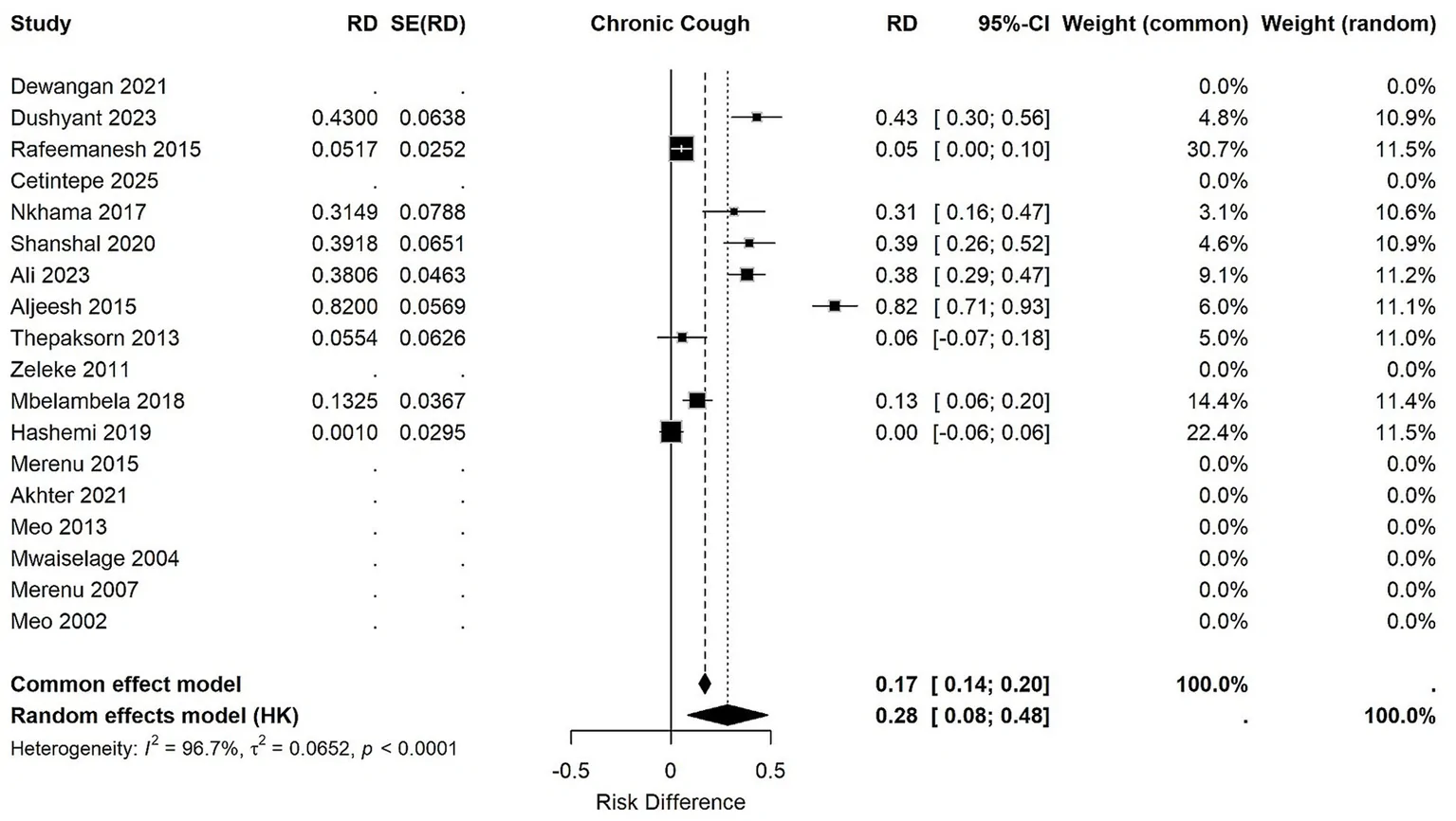
Forest plot for pooled risk differences (RD) in chronic cough reported within groups exposed and unexposed to cement dust from factories across nine eligible studies.

Among the four chronic respiratory symptoms reported by the participants exposed and unexposed to dust from cement factories, the absolute risk increase was statistically significant in case of chronic cough only. The absolute risk increase for exposed group was 28% higher (RD = 0.28; 95% CI: 0.08 to 0.48) as compared to unexposed group. However high heterogeneity (*I*^2^ = 96.7%) was observed (Figure 3).

The absolute increase for chronic wheeze, was statistically insignificant at 10% higher within exposed group (RD = 0.10; 95% CI: −0.01 to 0.20) as compared to unexposed group, with high heterogeneity (*I*^2^ = 85.7%) among studies (Supplementary Figure 3). Similarly, chronic phlegm, the absolute was statistically insignificant at 15% higher (RD = 0.15; 95% CI: −0.01 to 0.32) in exposed group as compared to unexposed group, with high heterogeneity (*I*^2^ = 90.9%) (Supplementary Figure 4). In case of chronic chest pain, the absolute risk showed non-significant increase in risk of 18% in exposed group (RD = 0.18; 95% CI: −0.10 to 0.46) as compared to unexposed group, with very high heterogeneity (*I*^2^ = 97.5%) (Supplementary Figure 5).

### 3.5 Sensitivity analyses

To assess the robustness of the pooled estimates and influence of individual studies on statistical assumptions, we performed sensitivity analyses using both the Hartung–Knapp adjustment and leave-one-out influence analyses. Following Hartung–Knapp adjustment, the pooled associations for FEV_1_ and FVC remained statistically significant, whereas the association for the FEV₁/FVC ratio was attenuated and was no longer statistically significant. Leave-one-out analyses further demonstrated that the pooled estimates were robust to the exclusion of individual studies. For FEV_1_, pooled SMDs ranged from −0.597 to −0.765, and for FVC from −0.453 to −0.614, with all estimates remaining statistically significant. For the FEV_1_/FVC ratio, pooled SMDs ranged from −0.365 to −0.631 and remained statistically significant after omission of all studies except Cetintepe (2025) (44), where the confidence interval included the null (*p* = 0.058). Although heterogeneity remained significant across all analyses, no single study materially altered the magnitude or direction of the pooled estimates for outcomes, supporting the robustness of the primary findings (Supplementary Table 2).

### 3.6 Correlation between lung function and respiratory symptoms

**Figure 4:**
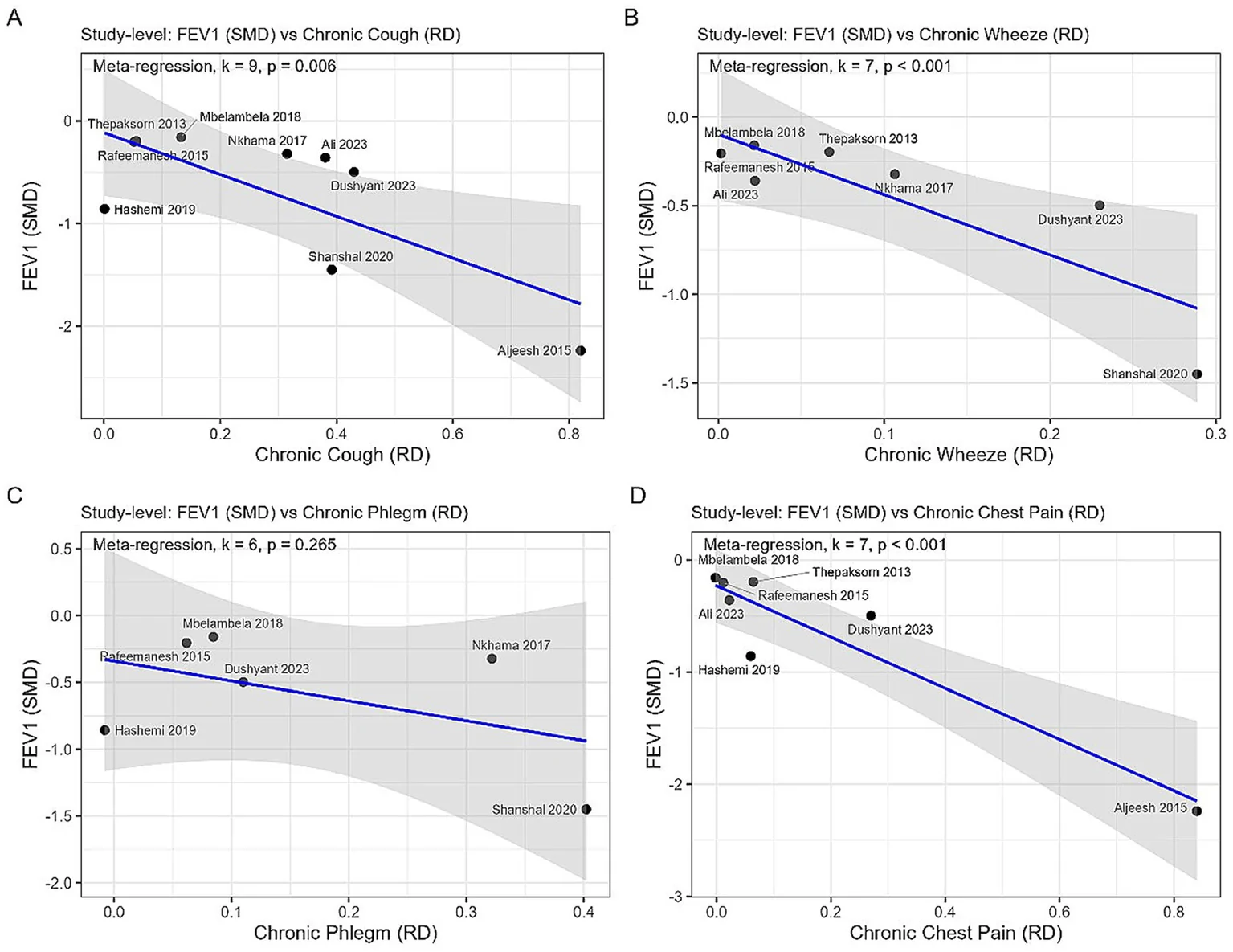
Study-level meta-regression of FEV_1_ (Standardized Mean Difference) against chronic respiratory symptom (Risk Difference). Meta-regression plots illustrate the relationship between FEV_1_ and **(A)** chronic cough, **(B)** chronic wheeze, **(C)** chronic phlegm, and **(D)** chronic chest pain.

**Figure 5:**
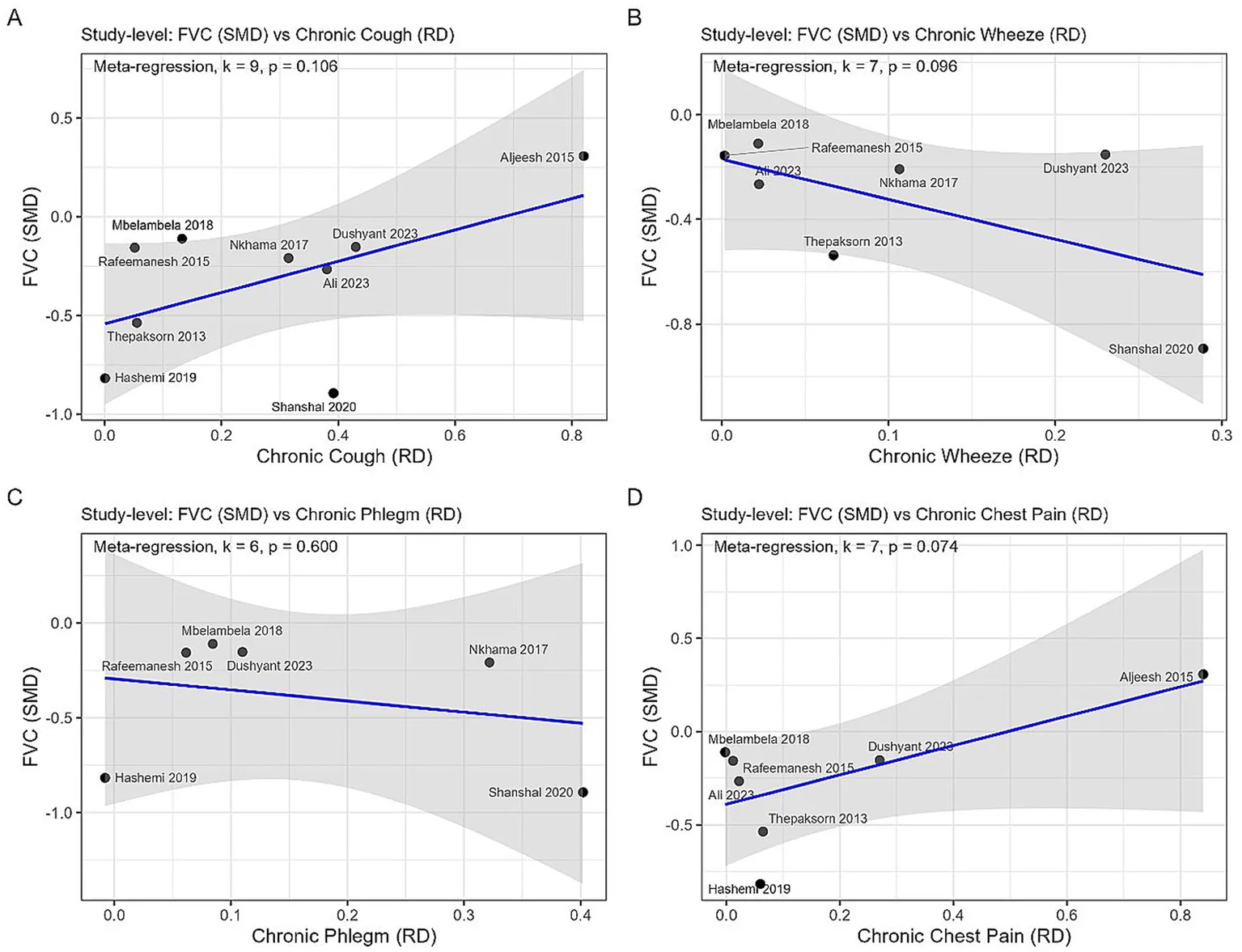
Study-level meta-regression of FVC (Standardized Mean Difference) against chronic respiratory symptoms (Risk Difference). Meta-regression plots illustrate the relationship between FVC and **(A)** chronic cough, **(B)** chronic wheeze, **(C)** chronic phlegm, and **(D)** chronic chest pain.

To assess the relationship between lung function parameters and chronic respiratory symptoms across studies reporting both, we performed an exploratory study level meta-regression analysis (Table 4). For FEV_1_, a significant inverse relationship was observed for chronic cough, wheeze and chest pain (Figure 4). Increasing risk difference for chronic cough was associated with a significant reduction in FEV₁ (*β* = −1.949, SE = 0.711, *p* = 0.006). Similarly, increasing risk differences of chronic wheeze (*β* = −3.321, SE = 0.954, *p* < 0.001) and chronic chest pain (*β* = −2.225, SE = 0.464, *p* < 0.001) were also associated with significant decrease in FEV_1_. The association between FEV_1_ and chronic phlegm remained insignificant. In contrast, FVC did not show statistically significant associations with any of the chronic respiratory symptoms (Figure 5).

**Table 4:** Meta-regression analysis examining the association between pooled standardized mean differences (SMD) for lung function parameters and pooled risk differences (RD) for respiratory symptoms.

Lung function parameters	Chronic respiratory symptoms	Studies (n)	*β* Coefficient	Standard Error	*p*-value
FEV1	Chronic cough	9	−1.949	0.711	0.006
	Chronic wheeze	7	−3.321	0.954	<0.001
	Chronic phlegm	6	−1.489	1.335	0.265
	Chronic chest pain	7	−2.225	0.464	<0.001
FVC	Chronic cough	9	0.764	0.473	0.106
	Chronic wheeze	7	−1.503	0.902	0.096
	Chronic phlegm	6	−0.578	1.102	0.6
	Chronic chest pain	7	0.775	0.434	0.074
FEV1/FVC ratio	Chronic cough	9	−4.648	1	<0.001
	Chronic wheeze	7	−2.684	0.853	0.002
	Chronic phlegm	6	−1.727	1.083	0.111
	Chronic chest pain	7	−5.09	0.702	<0.001

**Figure 6:**
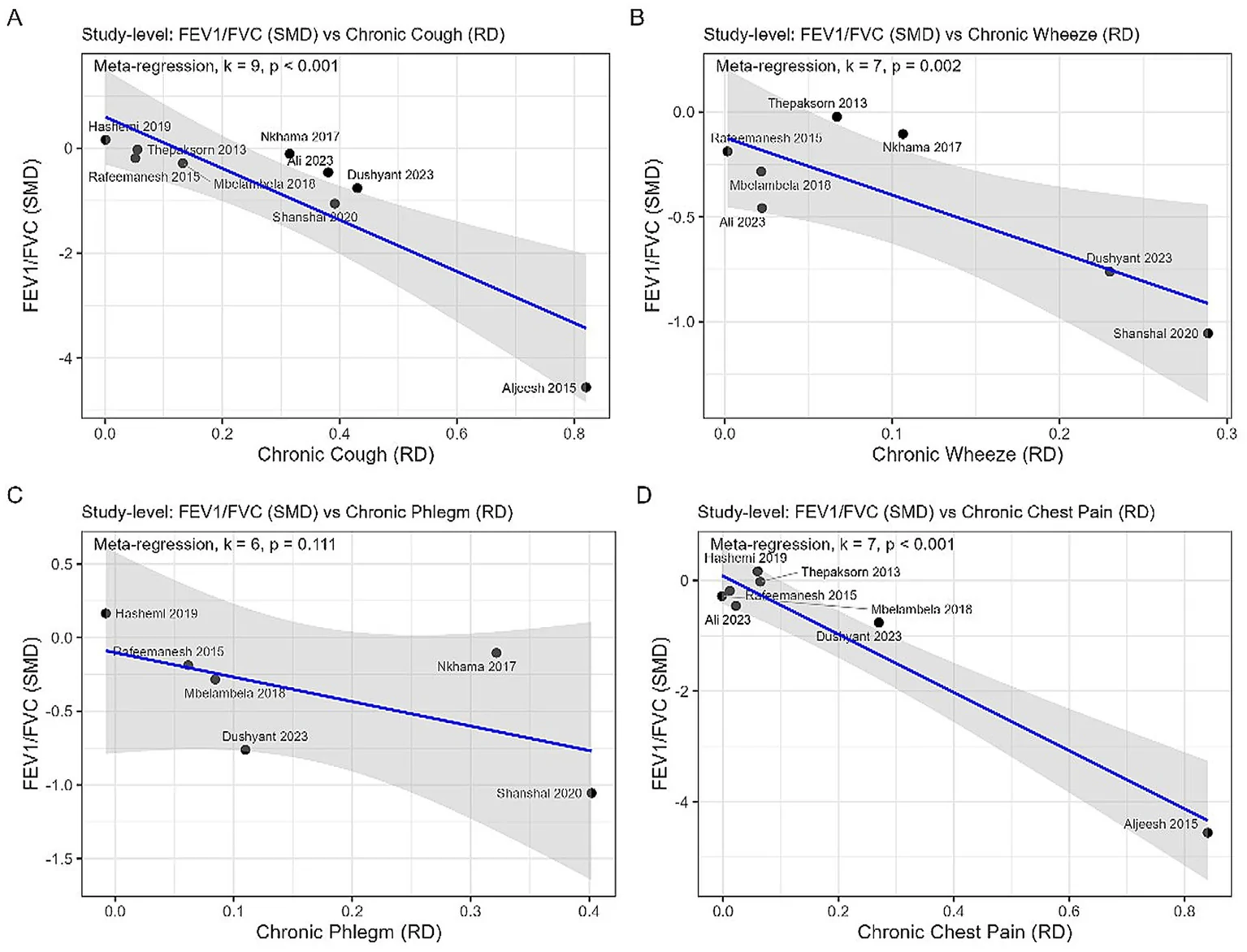
Study-level meta-regression of FEV₁/FVC ratio (Standardized Mean Difference) against chronic respiratory symptom (Risk Difference). Meta-regression plots illustrate the relationship between FEV₁/FVC ratio and **(A)** chronic cough, **(B)** chronic wheeze, **(C)** chronic phlegm, and **(D)** chronic chest pain.

For the FEV_1_/FVC ratio, a significant inverse relationship was observed for chronic cough, wheeze and chest pain. Increasing risk difference for chronic cough was associated with a significant reduction in FEV_1_/FVC ratio (*β* = −4.648, SE = 1.00, *p* < 0.001; Figure 6). Similarly, increasing risk difference of chronic wheeze (*β* = −2.684, SE = 0.853, *p* = 0.001) and chronic chest pain (*β* = −5.091, SE = 0.702, *p* < 0.001) were associated with a significant decrease in FEV_1_/FVC ratio. The association between FVC and chronic phlegm remained insignificant.

### 3.7 Subgroup analysis by world bank income classification

In the first step, we performed a subgroup analysis to compare the effect of dust from cement factories on the lung function parameters (FEV_1_, FVC and FEV_1_/FVC ratios) in the exposed and unexposed groups based on the socioeconomic status of the study population as defined by world bank income classification. The subgroup analysis included studies from 11 lower-middle income countries, followed by five upper-middle income countries and two lower-income countries. The effect of dust from cement factories on the lung function parameters within each socioeconomic classification were statistically insignificant (Table 5; Supplementary Table 3).

**Table 5:** Subgroup analysis of pooled standardized mean differences (SMD) for lung function outcomes (FEV_1_, FVC, and FEV_1_/FVC ratio) stratified by world bank income classification.

Lung Function Parameter	Income Group	Studies (n)	SMD	95% CI	Standard Error	*I*^2^ (%)	*τ* ^2^	Q Statistic	*p*-value
FEV1	Overall	17	−0.713	(−1.052, −0.375)	0.173	96.23	0.478	1.793	0.408
	LMIC	10	−0.861	(−1.381, −0.340)	0.266	96.97	0.672		
	UMIC	5	−0.679	(−1.126, −0.231)	0.228	94.16	0.244		
	LIC	2	−0.131	(−0.659, 0.397)	0.269	73.35	0.109		
FVC	Overall	18	−0.564	(−0.917, −0.211)	0.180	96.63	0.557	2.266	0.322
	LMIC	11	−0.503	(−0.968, −0.038)	0.237	96.56	0.588		
	UMIC	5	−0.916	(−1.581, −0.250)	0.340	97.18	0.560		
	LIC	2	−0.030	(−0.572, 0.512)	0.277	74.64	0.117		
FEV1/FVC Ratio	Overall	18	−0.573	(−1.076, −0.069)	0.257	98.34	1.158	0.114	0.945
	LMIC	11	−0.589	(−1.361, 0.182)	0.394	98.73	1.668		
	UMIC	5	−0.652	(−1.499, 0.195)	0.432	98.29	0.917		
	LIC	2	−0.406	(−0.624, −0.188)	0.111	0.00	0.000		

### 3.8 Subgroup analysis by type of exposure

Subgroup analyses based on mode of exposure demonstrated significant decline in lung function among both populations exposed through occupational and residential modes (Table 6). For FEV_1_, occupational exposure was associated with a larger reduction in lung function (SMD = −0.808, 95% CI: −1.210 to −0.406) compared with residential exposure (SMD = −0.263, 95% CI: −0.394 to −0.132). A similar pattern was observed for FVC, with occupational exposure showing a greater decline (SMD = −0.623, 95% CI: −1.046 to −0.199) than residential exposure (SMD = −0.315, 95% CI: −0.446 to −0.183). Likewise, the reduction in the FEV_1_/FVC ratio was more pronounced among occupationally exposed individuals (SMD = −0.631, 95% CI: −1.242 to −0.021) than among residentially exposed populations (SMD = −0.341, 95% CI: −0.473 to −0.209).

**Table 6:** Subgroup analysis of pooled standardized mean differences (SMD) for lung function parameters stratified by type of exposure (occupational vs. residential), with corresponding heterogeneity estimates.

Outcome	Subgroup	Studies (n)	SMD	95% CI	SE	I^2^ (%)	τ^2^	Q Statistic	df	p-value
FEV1	Overall	17	−0.713	(−1.052, −0.375)	0.173	96.23	0.478	1.320	1	0.251
	Occupational	14	−0.808	(−1.210, −0.406)	0.205	96.48	0.558			
	Residential	3	−0.263	(−0.394, −0.132)	0.067	0.00	0.000			
FVC	Overall	18	−0.564	(−0.917, −0.211)	0.180	96.63	0.557	0.486	1	0.486
	Occupational	15	−0.623	(−1.046, −0.199)	0.216	96.94	0.671			
	Residential	3	−0.315	(−0.446, −0.183)	0.067	0.00	0.000			
FEV1/FVC Ratio	Overall	18	−0.573	(−1.076, −0.069)	0.257	98.34	1.158	0.216	1	0.642
	Occupational	15	−0.631	(−1.242, −0.021)	0.312	98.52	1.423			
	Residential	3	−0.341	(−0.473, −0.209)	0.067	0.03	0.000			

Despite the consistently larger effect sizes observed among occupationally exposed workers, formal tests for subgroup differences did not demonstrate statistically significant differences between occupational and residential exposure for FEV_1_ (Q = 1.320, *p* = 0.251), FVC (Q = 0.486, *p* = 0.486), or the FEV_1_/FVC ratio (Q = 0.216, *p* = 0.642). These findings indicate that, although occupational exposure appears to be associated with greater impairment in lung function, the evidence was insufficient to conclude that the magnitude of effect differs significantly between occupational and residential exposure.

Heterogeneity remained substantial among occupational studies (*I*^2^ = 96.48–98.52%), reflecting considerable variability in study characteristics, exposure intensity, and participant populations. In contrast, heterogeneity was negligible among residential studies (*I*^2^ = 0.00–0.03%), suggesting consistent findings across the three residential studies included in the analysis.

## 4 Discussion

Exposure to dust from cement factories causes significant decline in lung function, this decline is marked by reduction in parameters namely; FEV_1_, FVC, and their ratio (FEV_1_/FVC ratio). This reduction in lung function and its parameters is attributed to a wide range of factors that include but are not limited to mode and duration of exposure before resulting in chronic symptoms (19, 43, 48, 49). To the best of our knowledge, this is the first systematic review and meta-analysis to evaluate the relationship between reduced lung function caused by exposure to emissions such as dust from the cement factories and chronic respiratory symptoms, considering both the mode of exposure and socio-economic status. Significant reduction in FEV_1_ and FVC indicates that occupational exposure to cement dust is associated with impaired pulmonary function, consistent to both the obstructive and restrictive patterns of respiratory impairment (24, 43). This association is biologically plausible, as the inhaled particulate matter causes chronic upper airway inflammation resulting in oxidative stress, worsening to structural remodeling of the respiratory tract, ultimately leading to progressive loss of pulmonary function (50–52).

Among the spirometric indices, FEV_1_ appeared more susceptible to exposure from cement dust, suggesting that airflow limitation as the earliest manifestation of decline in pulmonary function in exposed workers. As the respirable cement dust constitutes mostly of crystalline silica and alkaline irritants. These irritants penetrate the distal airways and trigger inflammatory and oxidative responses that promote narrowing of bronchi, hypersecretion of mucus, and airway remodeling, reducing expiratory airflow (53). The observed relationship between reduced FEV_1_ and chronic respiratory symptoms such as chronic cough, wheeze, and chest pain further reinforces the clinical utility of FEV_1_ as a sensitive indicator of early lung function impairment. The findings suggest that the increasing exposure and manifestation of chronic respiratory symptoms result in deterioration of pulmonary function (54).

Similarly, FVC levels were also reduced, although the magnitude of their effect was lower compared to FEV_1_. Reduced FVC may reflect restrictive physiological changes arising from chronic inflammation, parenchymal injury, or fibrotic remodeling following prolonged inhalation of mineral dust (55, 56). Unlike FEV_1_, the absence of relationship between FVC and chronic respiratory symptoms suggest the reduction in lung volume to remain silent, with no clinical manifestations during early stages of disease (57), where airway obstruction is more likely to result in respiratory complaints. The reduction in FEV_1_/FVC ratio further supports the presence of airflow obstruction among individuals exposed to dust from cement factories (58, 59). As the inflammation disproportionally impairs expiratory flow by narrowing bronchi and bronchioles, lung volumes remain unaffected during early stages of disease. For FEV_1_/FVC ratio the loss of significance following Hartung–Knapp adjustment was mainly due to intra-study variability rather than the absence of biological association.

For chronic respiratory symptoms chronic cough was only significantly higher among exposed individuals, while other symptoms remained insignificant. Despite elevated risks, non-significance of other chronic respiratory symptoms may be reasoned to report differences, variation in assessment tools, or limited statistical power. However, because spirometry provides an objective, quantitative measure of airway function, lung function parameters may capture sub-clinical impairment before respiratory symptoms become persistent. Chronic respiratory symptoms and lung function decline should be regarded as distinct but clinically relevant outcomes of cement dust exposure rather than two measures of the same effect. These findings align with prior research on cement dust exposure and reduced lung function (60, 61). Given that cement production and use are near-universal, and that protective measures such as engineering controls, PPE, and health surveillance are often weaker in LMIC settings, this occupational risk carries substantial relevance worldwide, particularly in lower-resourced regions (49).

Adverse respiratory effects due to cement dust are not only due to exposure but are also augmented by the context in which exposure occurs. Workers in occupational settings experienced greater declines in lung function than individuals with residential exposure, likely to reflect higher cumulative dust doses resulting from prolonged exposure durations and more frequent inhalation of respirable particles in the workplace (62, 63). The magnitude of this effect may be further modified by the socioeconomic status of the country, as lower- and middle-income settings often face challenges in implementing effective occupational health policies, engineering dust-control measures, environmental monitoring routines, and consistent use of personal protective equipment. Limited access to occupational health services and periodic respiratory surveillance may also delay the identification and management of early lung impairment, allowing cumulative respiratory damage to progress (64). In contrast, residential exposure generally occurs at lower and more consistent concentrations, resulting in less variable respiratory effects. The greater heterogeneity observed among occupational studies likely reflects differences in workplace regulations, job-specific exposure intensity, safety practices, and cumulative duration of exposure across industries and countries (65, 66).

The substantial heterogeneity identified across pooled analyses was expected given the diversity of the included studies. Variations in exposure assessment, cumulative dust exposure, participant characteristics, spirometry protocols, and adjustment for important confounders such as smoking and co-exposures are all likely contributors to the observed variability. Although sensitivity analyses demonstrated that individual studies influenced the magnitude of pooled estimates, the consistency in the direction of association supports the overall robustness of the evidence. Rather than representing a methodological weakness alone, this heterogeneity also reflects the wide range of real-world occupational environments in which cement dust exposure occurs, thereby enhancing the generalizability of the findings while emphasizing the need for standardized exposure assessment and reporting in future research.

This systematic review and meta-analysis provide evidence that chronic occupational exposure to cement dust adversely affects pulmonary function, with airflow limitation emerging as the predominant physiological abnormality. Together with the observed reduction in FVC, these findings indicate that both obstructive and restrictive respiratory changes may develop following prolonged exposure, although obstructive impairment appears to be more consistently demonstrated. Future studies employing standardized exposure assessment, longitudinal follow-up, and harmonized spirometric protocols are needed to better characterize exposure-response relationships. From an occupational health perspective, these findings underscore the importance of effective dust-control strategies, routine respiratory surveillance, and appropriate use of personal protective equipment to reduce the long-term burden of respiratory disease among cement factories workers.
