# Supplementary material for: Exposure to cement industry emissions and respiratory health outcomes: a global systematic review and meta-analysis from 2000 to 2025
Source: Front Public Health. 2026 Sep 3;14:1887654. doi: 10.3389/fpubh.2026.1887654 (PMC13581693; doi:10.3389/fpubh.2026.1887654)
Supplement: Supplementary file 1 [file Supplementary_file_1.docx]

**Title of the study:** Exposure to Cement Industry Emissions and Respiratory Outcomes: A Global Systematic Review and Meta-analysis from 2000 to 2025

# **Supplementary Tables**

**Supplementary Table 1:** Search strings used for the literature search across four databases (PubMed, Web of Science, Scopus, and Google Scholar).

| **PubMed** | ("Dust"[Mesh] OR "Construction Materials"[Mesh] OR "Occupational Exposure"[Mesh] OR "Inhalation Exposure"[Mesh] OR cement*[Title/Abstract] OR "cement dust"[Title/Abstract] OR "cement factory"[Title/Abstract] OR "cement factories"[Title/Abstract] OR "cement plant"[Title/Abstract] OR "cement plants"[Title/Abstract] OR "cement industry"[Title/Abstract] OR "cement kiln"[Title/Abstract] OR "Portland cement"[Title/Abstract] OR "cement worker"[Title/Abstract] OR "cement workers"[Title/Abstract]) AND ("Respiratory Tract Diseases"[Mesh] OR "Lung Diseases"[Mesh] OR "Forced Expiratory Volume"[Mesh] OR "Vital Capacity"[Mesh] OR "Pulmonary Disease, Chronic Obstructive"[Mesh] OR "Spirometry"[Mesh] OR "Cough"[Mesh] OR "Respiratory Sounds"[Mesh] OR "Dyspnoea"[Mesh] OR "Asthma"[Mesh] OR "Pneumoconiosis"[Mesh] OR respiratory*[Title/Abstract] OR pulmonary[Title/Abstract] OR lung*[Title/Abstract] OR "lung function"[Title/Abstract] OR spirometer*[Title/Abstract] OR "forced expiratory volume"[Title/Abstract] OR FEV1[Title/Abstract] OR "forced vital capacity"[Title/Abstract] OR FVC[Title/Abstract] OR "chronic obstructive pulmonary disease"[Title/Abstract] OR COPD[Title/Abstract] OR bronchitis*[Title/Abstract] OR asthma[Title/Abstract] OR cough*[Title/Abstract] OR wheeze*[Title/Abstract] OR dyspnoea[Title/Abstract] OR dyspnoea[Title/Abstract] OR "chest pain"[Title/Abstract] OR phlegm[Title/Abstract] OR pneumoconiosis[Title/Abstract]) |
| --- | --- |
| **Web of Science** | TS=(cement* OR "cement dust" OR "cement factor*" OR "cement plant*" OR "cement industry" OR "cement kiln*" OR "Portland cement" OR "cement worker*") AND TS=(respiratory* OR pulmonary OR lung* OR "lung function" OR spirometer* OR "forced expiratory volume" OR FEV1 OR "forced vital capacity" OR FVC OR "chronic obstructive pulmonary disease" OR COPD OR bronchitis* OR asthma OR cough* OR wheeze* OR dyspnoea OR dyspnoea OR "chest pain" OR phlegm OR pneumoconiosis) |
| **Scopus** | TITLE-ABS-KEY(cement* OR "cement dust" OR "cement factor*" OR "cement plant*" OR "cement industry" OR "cement kiln*" OR "Portland cement" OR "cement worker*") AND TITLE-ABS-KEY(respiratory* OR pulmonary OR lung* OR "lung function" OR spirometer* OR "forced expiratory volume" OR FEV1 OR "forced vital capacity" OR FVC OR "chronic obstructive pulmonary disease" OR COPD OR bronchitis* OR asthma OR cough* OR wheeze* OR dyspnoea OR dyspnoea OR "chest pain" OR phlegm OR pneumoconiosis) |
| **Google Scholar** | ("cement dust" OR "cement industry" OR "cement factory" OR "cement plant" OR "cement worker") AND (respiratory OR pulmonary OR "lung function" OR spirometry OR FEV1 OR COPD OR asthma OR cough) |

**Supplementary Table 2:** Risk of Bias Assessment for studies eligible for meta-analysis.

|  | **Dewangan 2021** | **Dushyant 2023** | **Rafeemanesh 2015** | **Cetintepe 2025** | **Nkhama 2017** | **Shanshal 2020** | **Ali 2023** | **Aljeesh 2015** | **Thepaksorn 2013** | **Zeleke 2011** | **Mbelambela 2018** | **Hashemi 2019** | **Merenu 2015** | **Akhter 2021** | **Meo 2013** | **Mwaiselage, 2005** | **Merenu 2007** | **Meo 2002** |
| --- | --- | --- | --- | --- | --- | --- | --- | --- | --- | --- | --- | --- | --- | --- | --- | --- | --- | --- |
| Were the criteria for inclusion in the sample clearly defined? |  |  |  |  |  |  |  |  |  |  |  |  |  |  |  |  |  |  |
| Were the study subjects and the setting described in detail? |  |  |  |  |  |  |  |  |  |  |  |  |  |  |  |  |  |  |
| Was the exposure measured  in a valid and reliable way? |  |  |  |  |  |  |  |  |  |  |  |  |  |  |  |  |  |  |
| Were objective, standard criteria used for measurement of the condition? |  |  |  |  |  |  |  |  |  |  |  |  |  |  |  |  |  |  |
| Were confounding factors identified? |  |  |  |  |  |  |  |  |  |  |  |  |  |  |  |  |  |  |
| Were strategies to deal with confounding factors stated? |  |  |  |  |  |  |  |  |  |  |  |  |  |  |  |  |  |  |
| Were the outcomes measured in a valid and reliable way? |  |  |  |  |  |  |  |  |  |  |  |  |  |  |  |  |  |  |
| Was appropriate statistical  analysis used? |  |  |  |  |  |  |  |  |  |  |  |  |  |  |  |  |  |  |
| Overall Risk of Bias |  |  |  |  |  |  |  |  |  |  |  |  |  |  |  |  |  |  |

**Supplementary Table 3:** Subgroup analysis of pooled standardized mean differences (SMD) for lung function parameters stratified by country income level as per world bank’s classification (low- and lower-middle-income [LMIC], upper-middle-income [UMIC], and low-income countries [LIC]), with corresponding heterogeneity estimates.

| Lung Function Parameters | Income Group | Studies (n) | SMD | 95% CI | p-value | I² (%) | τ² |
| --- | --- | --- | --- | --- | --- | --- | --- |
| FEV1 | LMIC | 10 | −0.861 | (−1.381, −0.340) | 0.001 | 96.97 | 0.672 |
|  | UMIC | 5 | −0.679 | (−1.126, −0.231) | 0.003 | 94.16 | 0.244 |
|  | LIC | 2 | −0.131 | (−0.659, 0.397) | 0.626 | 73.35 | 0.109 |
| FVC | LMIC | 11 | −0.503 | (−0.968, −0.038) | 0.034 | 96.56 | 0.588 |
|  | UMIC | 5 | −0.916 | (−1.581, −0.250) | 0.007 | 97.18 | 0.560 |
|  | LIC | 2 | −0.030 | (−0.572, 0.512) | 0.913 | 74.64 | 0.117 |
| FEV1/FVC Ratio | LMIC | 11 | −0.589 | (−1.361, 0.182) | 0.134 | 98.73 | 1.668 |
|  | UMIC | 5 | −0.652 | (−1.499, 0.195) | 0.132 | 98.29 | 0.917 |
|  | LIC | 2 | −0.406 | (−0.624, −0.188) | <0.001 | 0.00 | 0.000 |

**Supplementary Table 4:** Leave-one-out sensitivity analysis evaluating the influence of individual studies on the pooled effect estimates for FEV_1_, FVC, and FEV_1_/FVC ratio.

| Outcome | Study Removed | SMD | 95% CI | I² (%) | τ² | p-value |
| --- | --- | --- | --- | --- | --- | --- |
| FEV1 | Dewangan 2021 | -0.730 | (-1.091, -0.368) | 96.35 | 0.514 | <0.001 |
|  | Dushyant 2023 | -0.728 | (-1.089, -0.367) | 96.50 | 0.514 | <0.001 |
|  | Rafeemanesh 2015 | -0.746 | (-1.102, -0.391) | 96.36 | 0.497 | <0.001 |
|  | Cetintepe 2025 | -0.715 | (-1.078, -0.352) | 96.29 | 0.518 | <0.001 |
|  | Nkhama 2017 | -0.738 | (-1.096, -0.380) | 96.52 | 0.505 | <0.001 |
|  | Shanshal 2020 | -0.667 | (-1.013, -0.320) | 96.23 | 0.470 | <0.001 |
|  | Ali 2023 | -0.737 | (-1.096, -0.378) | 96.38 | 0.508 | <0.001 |
|  | Aljeesh 2015 | -0.620 | (-0.920, -0.321) | 95.09 | 0.347 | <0.001 |
|  | Thepaksorn 2013 | -0.747 | (-1.102, -0.392) | 96.32 | 0.497 | <0.001 |
|  | Zeleke 2011 | -0.765 | (-1.107, -0.423) | 96.25 | 0.460 | <0.001 |
|  | Mbelambela 2018 | -0.750 | (-1.104, -0.395) | 96.17 | 0.493 | <0.001 |
|  | Hashemi 2019 | -0.705 | (-1.067, -0.343) | 96.38 | 0.516 | <0.001 |
|  | Merenu 2015 | -0.747 | (-1.103, -0.392) | 96.05 | 0.497 | <0.001 |
|  | Akhter 2021 | -0.597 | (-0.860, -0.334) | 93.65 | 0.263 | <0.001 |
|  | Meo 2013 | -0.717 | (-1.078, -0.356) | 96.61 | 0.516 | <0.001 |
|  | Mwaiselage 2004 | -0.709 | (-1.071, -0.347) | 96.50 | 0.517 | <0.001 |
|  | Meo 2002 | -0.711 | (-1.072, -0.350) | 96.61 | 0.515 | <0.001 |
| FVC | Dewangan 2021 | -0.577 | (-0.952, -0.202) | 96.71 | 0.594 | 0.003 |
|  | Dushyant 2023 | -0.589 | (-0.961, -0.217) | 96.79 | 0.585 | 0.002 |
|  | Rafeemanesh 2015 | -0.589 | (-0.961, -0.216) | 96.77 | 0.585 | 0.002 |
|  | Cetintepe 2025 | -0.464 | (-0.773, -0.154) | 95.20 | 0.395 | 0.003 |
|  | Nkhama 2017 | -0.585 | (-0.958, -0.212) | 96.88 | 0.587 | 0.002 |
|  | Shanshal 2020 | -0.545 | (-0.918, -0.172) | 96.83 | 0.588 | 0.004 |
|  | Ali 2023 | -0.582 | (-0.956, -0.208) | 96.76 | 0.591 | 0.002 |
|  | Aljeesh 2015 | -0.614 | (-0.973, -0.254) | 96.66 | 0.545 | <0.001 |
|  | Thepaksorn 2013 | -0.566 | (-0.942, -0.190) | 96.81 | 0.597 | 0.003 |
|  | Zeleke 2011 | -0.611 | (-0.972, -0.251) | 96.72 | 0.549 | <0.001 |
|  | Mbelambela 2018 | -0.592 | (-0.963, -0.220) | 96.62 | 0.582 | 0.002 |
|  | Hashemi 2019 | -0.549 | (-0.924, -0.175) | 96.72 | 0.592 | 0.004 |
|  | Merenu 2015 | -0.577 | (-0.952, -0.201) | 96.57 | 0.595 | 0.003 |
|  | Akhter 2021 | -0.453 | (-0.744, -0.163) | 94.95 | 0.349 | 0.002 |
|  | Meo 2013 | -0.548 | (-0.921, -0.175) | 96.91 | 0.589 | 0.004 |
|  | Mwaiselage 2004 | -0.574 | (-0.950, -0.199) | 96.82 | 0.595 | 0.003 |
|  | Merenu 2007 | -0.611 | (-0.973, -0.250) | 96.65 | 0.551 | <0.001 |
|  | Meo 2002 | -0.529 | (-0.896, -0.162) | 96.81 | 0.569 | 0.005 |
| FEV1/FVC Ratio | Dewangan 2021 | -0.589 | (-1.125, -0.053) | 98.39 | 1.239 | 0.031 |
|  | Dushyant 2023 | -0.563 | (-1.098, -0.027) | 98.45 | 1.239 | 0.040 |
|  | Rafeemanesh 2015 | -0.597 | (-1.131, -0.062) | 98.43 | 1.232 | 0.029 |
|  | Cetintepe 2025 | -0.476 | (-0.968, 0.017) | 98.11 | 1.042 | 0.058 |
|  | Nkhama 2017 | -0.601 | (-1.134, -0.068) | 98.47 | 1.226 | 0.027 |
|  | Shanshal 2020 | -0.545 | (-1.078, -0.012) | 98.44 | 1.224 | 0.045 |
|  | Ali 2023 | -0.581 | (-1.117, -0.044) | 98.42 | 1.242 | 0.034 |
|  | Aljeesh 2015 | -0.365 | (-0.661, -0.069) | 95.15 | 0.364 | 0.016 |
|  | Thepaksorn 2013 | -0.606 | (-1.138, -0.074) | 98.40 | 1.221 | 0.026 |
|  | Zeleke 2011 | -0.596 | (-1.129, -0.062) | 98.50 | 1.230 | 0.029 |
|  | Mbelambela 2018 | -0.591 | (-1.127, -0.055) | 98.37 | 1.237 | 0.031 |
|  | Hashemi 2019 | -0.617 | (-1.146, -0.089) | 98.33 | 1.203 | 0.022 |
|  | Merenu 2015 | -0.588 | (-1.124, -0.052) | 98.31 | 1.239 | 0.032 |
|  | Akhter 2021 | -0.615 | (-1.144, -0.087) | 98.46 | 1.204 | 0.022 |
|  | Meo 2013 | -0.621 | (-1.147, -0.095) | 98.44 | 1.194 | 0.021 |
|  | Mwaiselage 2004 | -0.548 | (-1.082, -0.015) | 98.43 | 1.228 | 0.044 |
|  | Merenu 2007 | -0.597 | (-1.131, -0.063) | 98.46 | 1.231 | 0.028 |

# **Supplementary Figures**

**Supplementary Figure 1:** Forest plot of pooled Standard Mean Differences (SMD) for Forced Exhaled Volume in one second (FEV_1_) within groups exposed and unexposed to cement dust from factories across seventeen eligible studies.

**Supplementary Figure 2:** Forest plot of pooled Standard Mean Differences (SMD) for Forced Vital Capacity (FVC) within groups exposed and unexposed to cement dust from factories across eighteen eligible studies

**Supplementary Figure 3:** Forest plot for pooled Risk Differences (RD) in chronic wheeze reported within groups exposed and unexposed to cement dust from factories across seven eligible studies.

**Supplementary Figure 4:** Forest plot for pooled Risk Differences (RD) in chronic phlegm reported within groups exposed and unexposed to cement dust from factories across six eligible studies.

**Supplementary Figure 5:** Forest plot for pooled Risk Differences (RD) in chronic chest pain reported within groups exposed and unexposed to cement dust from factories across seven eligible studies.
